# Comprehensive Assessment of the Association between *FCGR*s polymorphisms and the risk of systemic lupus erythematosus: Evidence from a Meta-Analysis

**DOI:** 10.1038/srep31617

**Published:** 2016-08-19

**Authors:** Xiao-Wei Zhu, Yong Wang, Yi-Hua Wei, Pian-Pian Zhao, Xiao-Bo Wang, Jing-Jing Rong, Wen-Ying Zhong, Xing-Wei Zhang, Li Wang, Hou-Feng Zheng

**Affiliations:** 1 The Affiliated Hospital and Institute of Aging Research, School of Medicine, Hangzhou Normal University, Hangzhou, Zhejiang, China; 2 Department of Cardiovascular Medicine, the Second People’s Hospital of Wuhu City, Wuhu, Anhui, China; 3 Department of Dermatology, The Affiliated Hospital of Binzhou Medical University, Binzhou, Shandong, China

## Abstract

We performed a meta analysis to assess the relationship of FCGRs polymorphisms with the risk of SLE. Thirty-five articles (including up to 5741 cases and 6530 controls) were recruited for meta-analysis. The strongest association was observed between *FCGR2B* rs1050501 and SLE under the recessive genotypic model of C allele in the overall population (CC vs CT/TT, OR = 1.754, 95%CI: 1.422–2.165, P = 1.61 × 10^−7^) and in Asian population (CC vs CT/TT, OR = 1.784, 95%CI; 1.408–2.261, P = 1.67 × 10^−6^). We also found that *FCGR3A* rs396991 were significant association with the susceptibility to SLE in overall population in recessive model of T allele (TT vs TG/GG, OR = 1.263, 95%CI: 1.123–1.421, P = 9.62 × 10^−5^). The results also showed that significant association between *FCGR2A* rs1801274 and SLE under the allelic model in the overall population (OR = 0.879 per A allele, 95%CI: 0.819–0.943, P = 3.31 × 10^−4^). The meta-analysis indicated that *FCGR3B* copy number polymorphism NA1**·**NA2 was modestly associated with SLE in overall population (OR = 0.851 per NA1, 95%CI: 0.772–0.938, P = 1.2 × 10^−3^). We concluded that *FCGR2B* rs1050501 C allele and *FCGR3A* rs396991 T allele might contribute to susceptibility and development of SLE, and were under recessive association model. While, *FCGR2A* rs1801274 A allele and *FCGR3B* NA1 were associated with SLE and reduced the risk of SLE.

Systemic lupus erythematosus (SLE) is a kind of autoimmune disease with a strong genetic predisposition caused by complicated factors, it is also considered as an inflammatory disease caused by the mediation and deposition of immune complexes (ICs), leading to damage of multiple organs^1^. In different races or regions, the morbidity rate of SLE is quite different^2^,^3^, it is about 31-70/100,000 across China^4^, while it is 7-71/100,000 in Europeans^5^ and it increases to 200/100,000 in African population^5^. The etiology and pathogenesis of SLE is unclear yet, it is generally accepted that both genetic and environmental factors are involved in the development of this complex disease^6^. Since the end of last century, scientists were trying to use genetic linkage analysis to investigate the mechanism of SLE, a number of susceptibility area in SLE had been found such as 1q23^7^, 1q41^8^, 4p16^9^, 11q14^10^, 12q24^11^. Linkage analysis for SLE had made some achievements, but it is not easy to find real susceptibility genes because of large positioning areas. Then, candidate gene association studies (CGASs), in which single-nucleotide polymorphisms (SNPs) were assayed in cases and controls, were widely used and found some valuable susceptibility genes such as *IL-6*^12^, *TLR2*^13^, *VDR*^14^, *CTLA-4*^15^, *FCGR2A*^16^, *FCGR2B*^17^, *PELI1*^18^, *IKZF3*^19^. More recently, genome-wide association studies (GWAS) have been the powerful approach and found a lot of susceptibility genes and SNPs for SLE^20^,^21^,^22^,^23^,^24^,^25^,^26^,^27^.

Among these genes/proteins, FC gamma Receptor (*FCγR*) is a member of immunoglobulin superfamily, and it is very important to bind *FCγR* with the Fc protein of Immunoglobulin G (IgG), because *FCγR* binding may activate biological reaction, such as phagocytosis^28^. The human 1q21-23 locus contains 5 *FCGR* genes (*FCGR2A*, 2B, 2C, 3A and 3B) encoding the *FCγRII*and *FCγRIII* receptor**·**families^29^. FCγRs mediate clearance of immune complexes and have been strongly implicated in the pathogenesis of SLE and lupus nephritis^30^. Thus the genes that encode these receptors have been the focus of many genetic studies in SLE^31^.

*FCGR*s were not genome-wide significantly identified by any GWAS above, and the results were not always consistent by candidate gene association study. The inconsistency of findings is related to many factors, such as the selecting of the sample, the size of sample and the dealing of the statistics, etc. Therefore, in order to reduce the limitations of single study and to overcome the possible random errors, we performed a large-scale meta-analysis involving different ethnics. Among all the studies, there were 5082 cases and 4951 controls to evaluate the relationship between *FCGR2A* rs1801274 and SLE and there were 2970 cases and 4197 controls for *FCGR2B* rs1050501. For *FCGR3A* rs396991 and *FCGR3B* NA1**·**NA2, there were 5694 cases and 6450 controls, 1692 cases and 1899 controls, respectively. The purpose of this study is to analyze whether the polymorphisms of *FCGR*s are susceptibility to SLE. We also made efforts to find the best-fit association model among the additive, recessive and dominant models for the polymorphisms.

## Results

### Studies included in the meta-analysis

**Figure 1:**
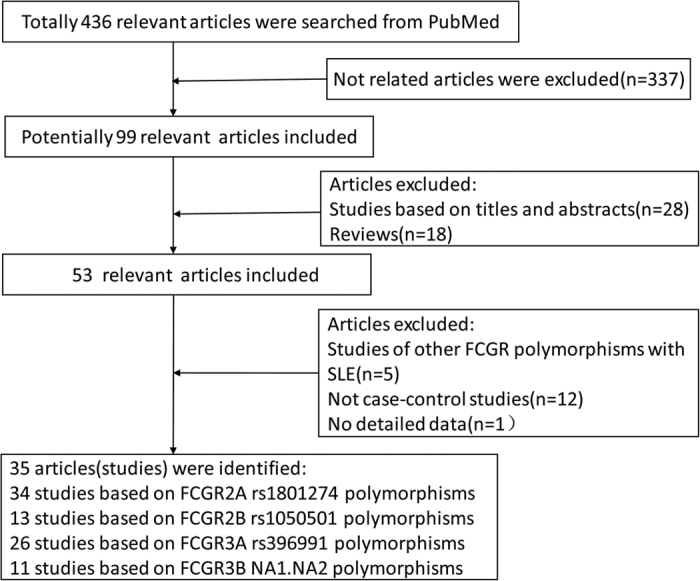
The process of the articles selected in this meta-analysis.

In this meta-analysis, totally 436 relevant articles were found from PubMed, of which 337 were excluded because they were unrelated articles. Studies investigating other *FCGR* gene polymorphisms were also excluded^17^,^32^,^33^,^34^,^35^,^36^. One more article was also excluded because there was no detail genotyping data^37^. After filtering, 35 eligible articles were finally included^16^,^33^,^38^,^39^,^40^,^41^,^42^,^43^,^44^,^45^,^46^,^47^,^48^,^49^,^50^,^51^,^52^,^53^,^54^,^55^,^56^,^57^,^58^,^59^,^60^,^61^,^62^,^63^,^64^,^65^,^66^,^67^,^68^,^69^. The flow chart of selecting articles process is presented in Fig. 1. Therefore, there were 34 studies with 5082 cases and 4951 controls to evaluate the relationship between *FCGR2A* rs1801274 polymorphism and SLE. For *FCGR2B* rs1050501 polymorphism, there were 13 studies involving a total of 2970 cases and 4197 controls. For *FCGR3A* rs396991 polymorphism and *FCGR3B* NA1**·**NA2 polymorphism, 26 studies (5694 cases and 6450 controls) and 11 studies (1692 cases and 1899 controls) were available, respectively. The basic information of these included studies genotype distributions and the allele frequencies are showed in Table 1.

### Meta-analysis results

#### *FCGR2A* rs1801274 polymorphism and SLE risk

**Figure 2:**
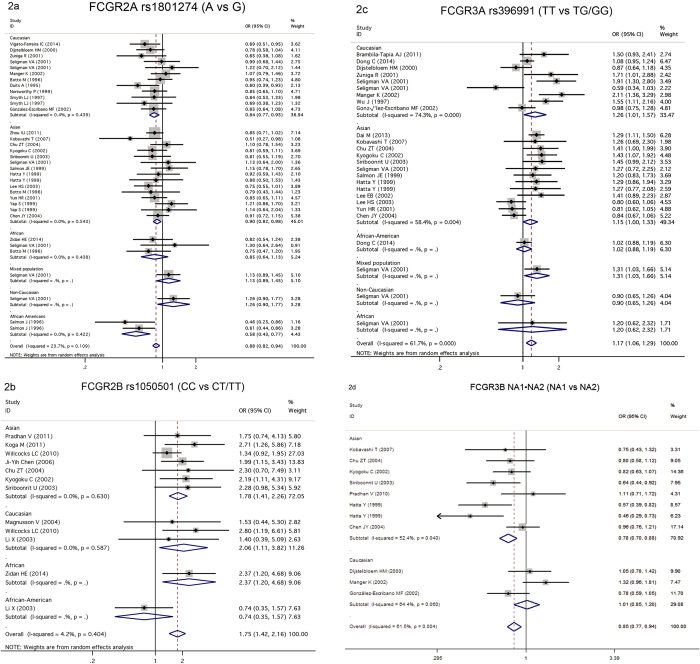
Forest plot for the meta-analysis of the association between FCGRs polymorphisms and SLE. (**a**) FCGR2A rs1801274 and SLE (A vs G); (**b**) *FCGR2B* rs1050501 and SLE (CC vs CT/TT); (**c**) FCGR3A rs396991 and SLE (TT vs TG /GG); (**d**) FCGR3B NA1**·**NA2 and SLE (NA1 vs NA2).

Test of heterogeneity in the overall population is not significant (P = 0.109, I2 = 23.70%), suggesting fixed effect model could be used. A strong association was found between rs1801274 and SLE under the allelic model in the overall population (OR = 0.879 per A allele, 95%CI: 0.819–0.943, P = 3.31 × 10^−4^, Table 2, Fig. 2a). Stratification analysis by ethnicity showed significant association between rs1801274 and SLE in Caucasian (OR = 0.845 per A allele, 95%CI: 0.766–0.932, P = 8.08 × 10^−4^, Table 2, Fig. 2a). And we also observed association between this polymorphism and SLE in African Americans (OR = 0.575 per A allele, 95%CI; 0.429–0.774, P = 2.73 × 10^−4^, Table 2, Fig. 2a) and in Asian population (OR = 0.896 per A allele, 95%CI: 0.822–0.977, P = 0.013, Table 2, Fig. 2a). No significant association was found in this meta-analysis between the polymorphism and the risk of SLE in African population (OR = 0.853 per A allele, 95%CI: 0.642–1.132, P = 0.271, Table 2, Fig. 2a). We also tested the dominant and recessive models of A allele in the overall, European, Asian and African populations, these results showed that the association was more significant in the recessive model than the dominant model in the overall population (Table 2, Supplementary Fig. S1a, Fig. S2a).

#### *FCGR2B* rs1050501 polymorphism and SLE risk

To assess the association of *FCGR2B* rs1050501 polymorphism with SLE, 13 studies were included in this meta-analysis with 2970 cases and 4197 controls, however, we identified publication bias while the study by Kobavashi T *et al*.^59^ was included (Begg’s Test: Z = 2.14, P = 0.033), therefore, this study was removed in the final analysis with 2899 cases and 4153 controls. After exclusion, the Begg’s test showed no deviation (Z = 1.58, P = 0.115) (Supplementary Table S1).

A very significant association was identified between rs1050501 and SLE under the recessive genotypic model of C allele in the overall population (CC vs CT/TT, OR = 1.754, 95%CI: 1.422–2.165, P = 1.61 × 10^−7^, Fig. 2b, Table 3) and in Asian population (CC vs CT/TT, OR = 1.784, 95%CI; 1.408–2.261, P = 1.67 × 10^−6^, Table 3, Fig. 2b), these associations were not significant under dominant model, suggesting the recessive association model was fit for rs1050501_C (Table 3). In allelic test model, Significant association between rs1050501 and SLE was identified in the overall population (OR = 1.236 per C allele, 95%CI: 1.069–1.429, P = 6.93 × 10^−3^, Table 3, Supplementary Fig. S2b), and in the Asian population (OR = 1.326 per C allele, 95%CI: 1.095–1.604, P = 6.14 × 10^−3^, Table 3, Supplementary Fig. S2b) and in African population (OR = 1.749 per C allele, 95%CI: 1.153–2.655, P = 8.54 × 10^−3^, Table 3, Supplementary Fig. S2b).

#### *FCGR3A* rs396991 polymorphism and SLE risk

There were 26 studies with 5694 cases and 6450 controls in our meta-analysis to evaluate the relationship between *FCGR3A* rs396991 polymorphism and SLE. Firstly, we tested the dominant and recessive models to estimate the relation between rs396991 and SLE risk (Table 4). We found that rs396991 were significant association with the susceptibility to SLE in overall population in recessive model of T allele (TT vs TG/GG, OR = 1.263, 95%CI: 1.123–1.421, P = 9.62 × 10^−5^, Table 4, Fig. 2c), and in Caucasian population (TT vs TG/GG, OR = 1.394, 95%CI: 1.087–1.789, P = 9.05 × 10^−3^) and in mixed population (TT vs TG/GG, OR = 1.585, 95%CI: 1.122–2.239, P = 9.05 × 10^−3^). Similarly, recessive model is the best fit for the association of rs396991_T, because we didn’t observe any association under dominant model in any populations (Table 4). We also tested the allelic model to observe the relationship between rs396991 and SLE. The significant association was seen between rs396991 and SLE in the overall population (OR = 1.17 per T allele, 95%CI: 1.059–1.291, P = 1.94 × 10^−3^, Table 4, Supplementary Fig. S2c). And we also found trend of association between this polymorphism and SLE in the stratified analysis of ethnicity: (Caucasian, OR = 1.259 per T allele, P = 0.039; Asian population, OR = 1.152 per T allele, P = 0.05, Table 4, Fig. 2c).

#### *FCGR3B* NA1**·**NA2 copy number polymorphism and SLE risk

Totally, 11 studies included 1692 cases and 1899 controls were in our meta-analysis to assess the relation between *FCGR3B* NA1**·**NA2 copy number polymorphism and SLE. The meta-analysis indicated that NA1**·**NA2 was modestly associated with SLE in overall population (allele genetic model: OR = 0.851 per NA1, 95%CI: 0.772–0.938, P = 1.2 × 10^−3^, Table 5, Fig. 2d; recessive model of NA1: OR = 0.799, 95%CI: 0.685–0.933, P = 0.005, Table 5, Supplementary Fig. S2d). Analysis by population showed that NA1**·**NA2 was modestly associated with SLE in Asian by three models (allele genetic model: OR = 0.785, 95%CI: 0.697–0.883, P = 6.07 × 10^−5^, Table 5, Fig. 2d; dominant model: OR = 0.684, 95%CI: 0.549–0.853, P = 7.2 × 10^−4^, Table 5, Supplementary Fig. S1d; recessive model: OR = 0.756, 95%CI: 0.635–0.898, P = 0.002, Table 5, Supplementary Fig. S2d).

### Allele frequency of the 3 SNPs and comparing to the 1000 genome population

In Table 6, we showed the distinct difference of allele frequencies in Asian, Caucasian, African and African American population in the meta-analysis of the 3 SNPs. The allele frequencies of the 3 SNPs in Asian, Caucasian, African and African American population in the meta–analysis were consistent with the allele frequencies in 1000 Genome Project EUR (European ancestry), ASN (Asian ancestry), AFR (African ancestry), ASW (Americans of African Ancestry), respectively.

### Publication bias and Sensitivity analysis

**Figure 3:**
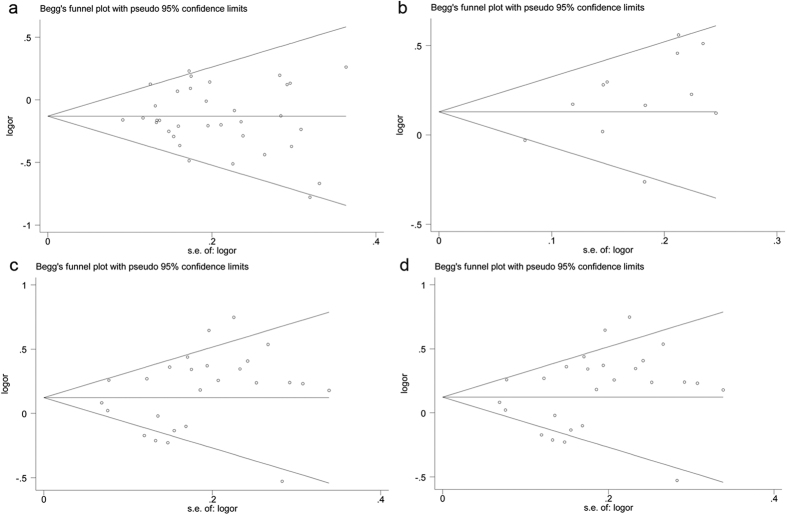
Begg’s funnel plot of publication bias in the meta-analysis of the association of FCGRs polymorphisms with SLE risk under allele genetic model. (**a**) FCGR2A rs1801274 and SLE (A vs G); (**b**) *FCGR2B* rs1050501 and SLE (C vs T); (**c**) FCGR3A rs396991 and SLE (T vs G); (**d**) FCGR3B NA1**·**NA2 and SLE (NA1 vs NA2).

**Figure 4:**
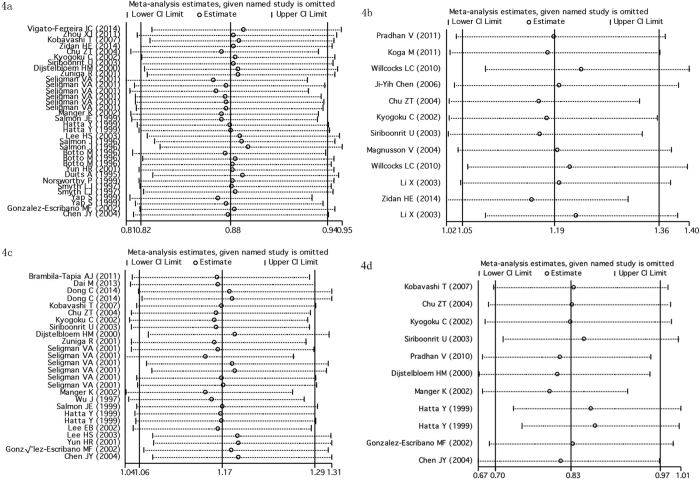
Sensitivity analysis to assess the stability of the meta-analysis. (**a**) FCGR2A rs1801274 in SLE; (**b**) *FCGR2B* rs1050501 in SLE; (**c**) FCGR3A rs396991 in SLE; (**d**) FCGR3B NA1**·**NA2 in SLE).

Begg’s funnel plot and Egger’s test were performed to estimate publication bias. There was no obvious evidence of symmetry from the shapes of the funnel plots (Fig. 3), and showed no evidence of publication bias in rs1801274 polymorphism (P = 0.594), rs396991 polymorphism (P = 0.252), NA1**·**NA2 polymorphism (P = 0.213), and rs1050501 polymorphism (P = 0.115, after excluded the study by Kobavashi T *et al*.^59^) under allele genetic model in our meta-analysis (Fig. 3a–d). We also conducted sensitivity analysis to assess the influence of individual studies on the pooled ORs. We found the pooled OR was not substantially altered, when any one study was deleted (Fig. 4a–d).

## Discussion

In this study, we conducted a meta-analysis of the association between *FCGR2A, 2B, 3A* and *3B* polymorphisms and SLE susceptibility. We found that C allele of rs1050501 (*FCGR2B*) and T allele of rs396991 (*FCGR3A*) strongly increase the risk of SLE. We also found significant association between *FCGR2A* rs1801274, *FCGR3B* copy number polymorphism NA1**·**NA2, and SLE in the overall population.

SNP rs1801274 is a missense mutation in *FCGR2A* gene on chromosome 1q23.3 (161479745), which encodes substitution of histidine (H) by arginine (R) in the IgG-binding domain of FcgRIIa and it was reported that FcgRIIa-R has a lower binding affinity for IgG than FcgRIIa-H^68^. In our study, we found *FCGR2A* rs1801274 contributes to SLE susceptibility in overall population. And in the subgroup analysis, the polymorphism was associated with SLE in Asian, Caucasian, and African Americans but not in African population, however, there were only 3 studies for African population in this meta-analysis, consisting only 190 cases and 198 controls, and the effect direction of A allele in African population is the same as that in the overall population. Previous study such as by Karassa FB *et al*.^70^ presented the association between *FCGR2A* rs1801274 and SLE of Caucasian descent, but it was less clear in subjects of Asian or African descent. Another study^71^ found a significant association of rs1801274 G allele and increased SLE risk in all groups, and a clear effect of G allele on SLE was shown in European and Asian, these results were consistent with our study. We also confirmed the findings from Zhou XJ^65^ that investigated the association between rs1801274 and SLE in Chinese population. In many ways, we suggest that rs1801274 was associated with SLE, especially in Caucasian and Asian population. As for other populations, more studies were needed to evaluate association between the polymorphism and SLE. It’s likely that such differences may, at less in part, be attributable to the ethnic difference.

GWAS have found that there were significant associations between *FCGR2A* rs1801274 and Kawasaki disease^72^ and Inflammatory bowel disease (P = 2.12 × 10^−38^, OR = 1.12)^73^ and there were genome-wide significant associations between the SNP and Ulcerative colitis in European^74^, and Japanese population^75^. There was only one genome-wide association study between *FCGR2A* and SLE, however, SNP rs1801274 was not genome-wide significant^27^.

FcgRIIb is an inhibitory receptor mediating B-cell function via an immune receptor tyrosine-based inhibitory motif 59. FcgRIIb is the only FcgR that transmits an inhibitory signal and is expressed in B cells and myelomonocytic cells^57^. *FCGR2B* rs1050501 (c.695T > C) codes a non-synonymous substitution, Ile232Thr (I232T) on chromosome 1q23.3 (161644048), our meta-analysis showed that C allele significantly increased the risk of SLE under recessive association model and allelic test model in overall population (Table 3, Fig. S2a; Supplementary Fig. S2b). By subgroup analysis, the association was also found under allelic genetic model and recessive model in Asian populations, but not in Caucasians under allelic genetic model. In 2004, Chu ZT *et al*.^57^ had found rs1050501 was significant associated with SLE in Chinese population. These results were in agreement with Lee YH *et al*.^76^ that indicated the C allele significantly increased the risk of SLE in Asian population. Therefore, it was suggested that the association between *FCGR2B* rs1050501 and SLE was on the basis of ethnicity, and the C allele is a risk for SLE in Asian.

FcγRIIIa is expressed on the surfaces of natural killer (NK) cells, monocytes and macrophages and binds to IgG1 and IgG3 subclasses^66^. *FCGR3A* rs396991 is a missense mutation on chromosome 1q23.3 (161514792), leading to a valine (V) substitution for phenylalanine (F) at amino acid residue 176 (including the leader sequence)^66^. In our meta-analysis, it suggested that a significant association between *FCGR3A* rs396991_T and SLE in overall population under recessive association models and allele genetic model (Table 4, Fig. 2c; Supplementary Fig. S2c). Previous study^77^ had suggested a modest trend of SLE predisposition for *FCGR3A* rs396991 in 1,261 SLE patients and 1,455 disease-free controls but with significant between-study heterogeneity. In addition, we observed trend of association between this polymorphism and SLE in the stratified analysis of ethnicity in Caucasian and Asian population, which was consistent with the study of Li *et al*.^78^. However, the association was not confirmed in the population of African and African American.

The copy number variation (NA1**·**NA2) in *FCGR3B* has shown to influence the interaction between FcγRIIIb and human IgG^61^. Individuals who are homozygous for NA1 allele has greater phagocytosis of IgG opsonized targets than that of NA2 homozygous individuals. Our meta-analysis illustrated a modest association between this copy number polymorphism and SLE in overall population by allele genetic model and recessive model. Analysis by population showed that NA1**·**NA2 was associated with SLE in Asians by three models. This association was not observed in a small sample size of 165 Chinese patients with SLE and 129 healthy controls by Chu ZT *et al*.^57^. To further explain the differences, we compared frequency between our meta analysis and those from Chu ZT *et al*.^57^ in Table 1, From this table, we could tell the frequencies were consistent between the two, the sample size might have been responsible for the different results. Besides, we didn’t find an association between *FCGR3B* NA1**·**NA2 polymorphism and SLE in Caucasian.

Though we tried to control the potential bias of publications and populations. There were still have several limitations to be taken into consideration in this meta-analysis. Firstly, although the overall sample size is large, the size of each study is relatively small, with the smallest sample of 30 cases and 31 controls. Secondly, the meta-analysis for ethnicity included data more from population with Caucasian and Asian origin, and the findings are applicable to only these populations, more studies are required in other populations. Furthermore, the mechanism of SLE is considered to be sophisticated, including gene-gene and gene-environment interactions. More studies with enough statistical power are needed for deeply evaluation. Lastly, publication bias might affect the results, because the studies that found any negative results may not have been published.

Despite the limitations, this meta-analysis illustrated that C allele of *FCGR2B* rs1050501 and T allele of *FCGR3A* rs396991 might contribute to susceptibility and development of SLE, and were under recessive association model. While, A allele of *FCGR2A* rs1801274 and *FCGR3B* NA1 were associated with SLE and reduced the risk of SLE. Considering the limited samples in Africans and African Americans in this meta-analysis, studies with larger sample size including diverse ethnic populations are still required to investigate the association between *FCGR*s genes polymorphisms and SLE in the future.

## Methods

### Identification of eligible studies

We aimed to analyze the association between *FCGR2A* (SNP rs1801274), *FCGR2B* (SNP rs1050501), *FCGR3A* (SNP rs396991), *FCGR3B* copy number polymorphism (NA1/NA2) polymorphisms and SLE. Therefore, all published literatures before December 2015 that investigated the association between these polymorphisms and SLE risk were searched using the PubMed engine (National Center for Biotechnology, National Library of Medicine). We looked for the articles with keywords “*FCGR2A*”, “*FCGR2B*”, “*FCGR3A*”, “*FCGR3B*”, “*FCγR*s”, “polymorphism” in combination with “Systemic Lupus erythematosus” or “SLE”. Finally, we extracted data from the published articles, not from conference abstracts or any meetings.

### Data extraction

All studies should meet the following conditions: 1) case-control study; 2) with original data to calculate genotype counts and odds ratio (OR); 3) the diagnosis of SLE patients according to the American College of Rheumatology criteria^79^,^80^. The following information is shown in our study: first author, year of publication, ethnicity, sample size of cases and controls, allele frequency and genotype frequency.

### Statistical analysis

The allele frequencies of polymorphisms from each study were calculated by the allele counting method. Pooled ORs and 95% confidence intervals (CIs) were used to evaluate the strength of association between polymorphisms and SLE risk for every eligible study. Heterogeneity was evaluated using the I^2^ metric, which ranges between 0 and 100% (25%, low heterogeneity; 50%, moderate; 75%, high heterogeneity)^81^. If the P value for heterogeneity test was higher than 0.01, the fixed effect model was used to weight of each study. Moreover, the random effect model was also used. In this meta-analysis, P value of less than 0.05 was considered a statistically significant.

In order to get better search results, we evaluated possible publication bias by Egger’s linear regression text^82^. P value < 0.05 was considered representative of statistical publication bias^82^. We also used a funnel plot to evaluate the publication bias by Begg’s test^83^. For sensitivity analysis, removed one study from the total and tested residual studies. Statistical analysis was carried out using the software program STATA10.1 (Stata Corporation, College Station, Texas).

## Additional Information

**How to cite this article**: Zhu, X.-W. *et al*. Comprehensive Assessment of the Association between *FCGR*s polymorphisms and the risk of systemic lupus erythematosus: Evidence from a Meta-Analysis. *Sci. Rep*. **6**, 31617; doi: 10.1038/srep31617 (2016).

## Supplementary Material

Supplementary Information

## Figures and Tables

**Table 1:** The basic information of every studies included in this meta-analysis.

Polymorphismsand study	Journal	Year	Ethnicity	Sample size		Genotypes						Allele frequencies (%)			
				Cases	Controls	Cases			Controls			Cases		Controls	
**rs1801274(FCGR2A)**						**AA**	**AG**	**GG**	**AA**	**AG**	**GG**	**A**	**G**	**A**	**G**
Vigato-Ferreira IC	Autoimmunity	2014	Caucasian	157	160	23	59	75	35	43	82	0.334	0.666	0.353	0.647
Dijstelbloem HM	Arthritis Rheum	2000	Caucasian	230	154	54	108	68	42	80	32	0.470	0.530	0.532	0.468
Zuñiga R	Arthritis Rheum	2001	Caucasian	67	53	5	39	23	11	28	14	0.366	0.634	0.472	0.528
Seligman VA	Arthritis Rheum	2001	Caucasian	76	186	10	49	17	28	114	44	0.454	0.546	0.457	0.543
Seligman VA	Arthritis Rheum	2001	Caucasian	48	55	7	29	12	10	24	21	0.448	0.552	0.400	0.600
Manger K	Ann Rheum Dis	2002	Caucasian	140	187	46	55	39	53	84	50	0.525	0.475	0.508	0.492
Botto M	Clin Exp Immunol	1996	Caucasian	215	259	46	97	72	57	120	82	0.440	0.560	0.452	0.548
Duits A	Arthritis Rheum	1995	Caucasian	95	69	18	50	27	22	36	11	0.453	0.547	0.580	0.420
Norsworthy P	Arthritis Rheum	1999	Caucasian	195	283	32	96	67	62	131	90	0.410	0.590	0.451	0.549
Smyth LJ	Ann Rheum Dis	1997	Caucasian	81	66	10	49	22	12	38	16	0.426	0.574	0.470	0.530
Smyth LJ	Ann Rheum Dis	1997	Caucasian	42	52	14	16	12	20	24	8	0.524	0.476	0.615	0.385
González-Escribano MF	Eur J Immunogenet	2002	Caucasian	276	194	64	137	75	59	86	49	0.480	0.520	0.526	0.474
Zhou XJ	Lupus	2011	Asian	589	477	238	269	82	209	220	48	0.632	0.368	0.669	0.331
Kobavashi T	J Periodontol	2007	Asian	71	44	34	31	6	28	16	0	0.697	0.303	0.818	0.182
Chu ZT	Tissue Antigens	2004	Asian	163	129	72	70	21	53	58	18	0.656	0.344	0.636	0.364
Kyogoku C	Arthritis Rheum	2002	Asian	193	303	113	72	8	197	95	11	0.772	0.228	0.807	0.193
Siriboonrit U	Tissue Antigens	2003	Asian	87	187	37	40	10	93	76	18	0.655	0.345	0.701	0.299
Seligman VA	Arthritis Rheum	2001	Asian	57	40	11	37	9	6	27	7	0.518	0.482	0.488	0.513
Salmon JE	Arthritis Rheum	1999	Asian	148	97	70	66	12	41	47	9	0.696	0.304	0.665	0.335
Hatta Y	Genes Immun	1999	Asian	81	217	49	30	2	139	71	7	0.790	0.210	0.804	0.196
Hatta Y	Genes Immun	1999	Asian	69	93	42	26	1	62	28	3	0.797	0.203	0.817	0.183
Lee HS	Rheumatology	2003	Asian	299	144	131	114	54	67	66	11	0.629	0.371	0.694	0.306
Botto M	Clin Exp Immunol	1996	Asian	46	49	18	23	5	24	20	5	0.641	0.359	0.694	0.306
Yun HR	Lupus	2001	Asian	300	197	132	114	54	82	99	16	0.630	0.370	0.668	0.332
Yap S	Lupus	1999	Asian	175	108	59	91	25	28	63	17	0.597	0.403	0.551	0.449
Yap S	Lupus	1999	Asian	50	50	20	26	4	21	21	8	0.660	0.340	0.630	0.370
Chen JY	Ann Rheum Dis	2004	Asian	329	311	125	155	49	130	144	37	0.616	0.384	0.650	0.350
Zidan HE	Mol Biol Rep	2014	African	90	90	20	45	25	22	50	18	0.472	0.528	0.522	0.478
Seligman VA	Arthritis Rheum	2001	African	30	31	9	12	9	6	15	10	0.500	0.500	0.435	0.565
Botto M	Clin Exp Immunol	1996	African	70	77	8	37	25	17	35	25	0.379	0.621	0.448	0.552
Seligman VA	Arthritis Rheum	2001	mixed population	216	318	38	131	47	50	185	83	0.479	0.521	0.448	0.552
Seligman VA	Arthritis Rheum	2001	Non-Caucasian	140	132	28	82	30	22	71	39	0.493	0.507	0.436	0.564
Salmon J	J Clin Invest	1996	African Americans	43	39	4	23	16	14	15	10	0.360	0.640	0.551	0.449
Salmon J	J Clin Invest	1996	African Americans	214	100	37	97	80	27	50	23	0.400	0.600	0.520	0.480
**rs1050501(FCGR2B)**						**CC**	**CT**	**TT**	**CC**	**CT**	**TT**	**C**	**T**	**C**	**T**
Pradhan V	Indian J Med Res	2011	Asian	80	80	16	49	15	10	52	18	0.506	0.494	0.450	0.550
Koga M	J Hum Genet	2011	Asian	282	222	29	103	150	9	85	128	0.285	0.715	0.232	0.768
Willcocks LC	PNAS	2010	Asian	819	1026	60	284	475	57	404	565	0.247	0.753	0.252	0.748
Kobavashi T	J Periodontol	2007	Asian	71	44	4	26	41	0	6	38	0.239	0.761	0.068	0.932
Ji-Yih Chen	Arthritis Rheum	2006	Asian	351	372	39	123	189	22	144	206	0.286	0.714	0.253	0.747
Chu ZT	Tissue Antigens	2004	Asian	108	85	11	48	49	4	30	51	0.324	0.676	0.224	0.776
Kyogoku C	Arthritis Rheum	2002	Asian	193	303	21	66	106	16	104	183	0.280	0.720	0.224	0.776
Siriboonrit U	Tissue Antigens	2003	Asian	79	165	12	29	38	12	56	97	0.335	0.665	0.242	0.758
Magnusson V	Arthritis Rheum	2004	Caucasian	263	228	7	67	189	4	53	171	0.154	0.846	0.134	0.866
Willcocks LC	PNAS	2010	Caucasian	326	1296	9	48	269	13	232	1051	0.101	0.899	0.100	0.900
Li X	Arthritis Rheum	2003	Caucasian	148	137	6	30	112	4	27	106	0.142	0.858	0.128	0.872
Zidan HE	Mol Biol Rep	2014	African	90	90	32	39	19	17	44	29	0.572	0.428	0.433	0.567
Li X	Arthritis Rheum	2003	African-American	160	149	14	49	97	17	53	79	0.241	0.759	0.292	0.708
**rs396991(FCGR3A)**						**TT**	**TG**	**GG**	**TT**	**TG**	**GG**	**T**	**G**	**T**	**G**
Brambila-Tapia AJ	Rheumatol Int	2011	Caucasian	94	98	61	5	28	52	8	38	0.676	0.324	0.571	0.429
Dong C	Arthritis Rheumatol	2014	Caucasian	834	1185	392	370	72	517	564	104	0.692	0.308	0.674	0.326
Dijstelbloem HM	Arthritis Rheum	2000	Caucasian	230	154	92	108	30	66	73	15	0.635	0.365	0.666	0.334
Zuñiga R	Arthritis Rheum	2001	Caucasian	67	53	25	38	4	15	26	12	0.657	0.343	0.528	0.472
Seligman VA	Arthritis Rheum	2001	Caucasian	78	207	37	30	11	55	102	50	0.667	0.333	0.512	0.488
Seligman VA	Arthritis Rheum	2001	Caucasian	55	57	25	15	15	30	21	6	0.591	0.409	0.711	0.289
Manger K	Ann Rheum Dis	2002	Caucasian	140	187	55	64	21	62	75	50	0.621	0.379	0.532	0.468
Wu J	J Clin Invest	1997	Caucasian	200	113	87	92	21	29	69	15	0.665	0.335	0.562	0.438
González-Escribano MF	Eur J Immunogenet	2002	Caucasian	276	194	101	131	44	66	104	24	0.603	0.397	0.608	0.392
Dai M	Int J Rheum Dis	2013	Asian	732	886	376	308	48	381	427	78	0.724	0.276	0.671	0.329
Kobavashi T	J Periodontol	2007	Asian	71	44	43	22	6	24	15	5	0.761	0.239	0.716	0.284
Chu ZT	Tissue Antigens	2004	Asian	163	129	76	74	13	48	63	18	0.693	0.307	0.616	0.384
Kyogoku C	Arthritis Rheum	2002	Asian	193	303	110	76	7	145	132	26	0.767	0.233	0.696	0.304
Siriboonrit U	Tissue Antigens	2003	Asian	87	187	42	35	10	64	96	27	0.684	0.316	0.599	0.401
Seligman VA	Arthritis Rheum	2001	Asian	59	41	22	29	8	12	22	7	0.619	0.381	0.561	0.439
Salmon JE	Arthritis Rheum	1999	Asian	148	97	44	81	23	19	64	14	0.571	0.429	0.526	0.474
Hatta Y	Genes Immun	1999	Asian	81	217	43	34	4	100	99	18	0.741	0.259	0.689	0.311
Hatta Y	Genes Immun	1999	Asian	69	93	37	29	3	46	38	9	0.746	0.254	0.699	0.301
Lee EB	Rheum Int	2002	Asian	145	75	89	51	5	40	29	6	0.790	0.210	0.727	0.273
Lee HS	Rheumatology	2003	Asian	299	144	90	163	46	52	77	15	0.574	0.426	0.628	0.372
Yun HR	Lupus	2001	Asian	300	197	90	164	46	71	104	22	0.573	0.427	0.624	0.376
Chen JY	Ann Rheum Dis	2004	Asian	302	311	119	138	45	133	146	32	0.623	0.377	0.662	0.338
Dong C	Arthritis Rheumatol	2014	African-American	648	953	289	283	76	413	431	109	0.664	0.336	0.659	0.341
Seligman VA	Arthritis Rheum	2001	mixed population	233	348	97	96	40	108	172	68	0.622	0.378	0.557	0.443
Seligman VA	Arthritis Rheum	2001	Non-Caucasian	155	141	60	66	29	53	70	18	0.600	0.400	0.624	0.376
Seligman VA	Arthritis Rheum	2001	African	35	36	11	19	5	7	25	4	0.586	0.414	0.542	0.458
**NA1/NA2**						**NA1·NA1**	**NA1·NA2**	**NA2·NA2**	**NA1·NA1**	**NA1·NA2**	**NA2·NA2**	**NA1**	**NA2**	**NA1**	**NA2**
Kobavashi T	J Periodontol	2007	Asian	71	44	20	46	5	20	19	5	0.606	0.394	0.670	0.330
Chu ZT	Tissue Antigens	2004	Asian	163	129	46	90	29	41	74	14	0.552	0.448	0.605	0.395
Kyogoku C	Arthritis Rheum	2002	Asian	193	303	62	98	33	116	145	42	0.575	0.425	0.622	0.378
Siriboonrit U	Tissue Antigens	2003	Asian	87	187	30	39	18	85	82	20	0.569	0.431	0.674	0.326
Pradhan V	Int J Rheum Dis	2010	Asian	80	80	20	32	28	18	32	30	0.450	0.550	0.425	0.575
Hatta Y	Genes Immun	1999	Asian	81	217	23	38	20	92	100	25	0.519	0.481	0.654	0.346
Hatta Y	Genes Immun	1999	Asian	69	93	18	33	18	44	39	10	0.500	0.500	0.683	0.317
Chen JY	Ann Rheum Dis	2004	Asian	302	311	117	132	53	119	145	47	0.606	0.394	0.616	0.384
Dijstelbloem HM	Arthritis Rheum	2000	Caucasian	230	154	42	101	87	27	66	61	0.402	0.598	0.390	0.610
Manger K	Ann Rheum Dis	2002	Caucasian	140	187	13	87	40	20	87	80	0.404	0.596	0.340	0.660
González-Escribano MF	Eur J Immunogenet	2002	Caucasian	276	194	30	77	169	20	75	99	0.248	0.752	0.296	0.704

**Table 2:** Meta-analysis of the association between FCGR2A rs1801274 polymorphism and SLE risk.

Population	N	A vs. G(allele model)			AA vs. AG+GG(recessive model)			AA+AG vs. GG(dominant model)		
		OR(95%CI)	P_OR_	P_h_	OR(95%CI)	P_OR_	P_h_	OR(95%CI)	P_OR_	P_h_
Overall	34	0.879(0.819–0.943)	3.31 × 10^−4^	0.109	0.867(0.784–0.960)	6.14 × 10^−3^	0.214	0.843(0.739–0.961)	0.011	0.074
Caucasian	12	0.845(0.766–0.932)	8.08 × 10^−4^	0.439	0.775(0.655–0.917)	3.08 × 10^−3^	0.522	0.883(0.756–1.032)	0.117	0.427
Asian	15	0.896(0.822–0.977)	0.013	0.543	0.932(0.830–1.046)	0.232	0.658	0.767(0.604–0.975)	0.030	0.179
African	3	0.853(0.642–1.132)	0.271	0.438	0.836(0.428–1.633)	0.601	0.192	0.802(0.515–1.250)	0.331	0.688
Mixed population	1	1.133(0.887–1.448)	0.318	—	1.144(0.721–1.817)	0.568	—	1.27(0.844–1.911)	0.252	—
Non-Caucasian	1	1.259(0.898–1.765)	0.181	—	1.250(0.674–2.317)	0.479	—	1.538(0.887–2.666)	0.125	—
African Americans	2	0.575(0.427–0.774)	2.73 × 10^−4^	0.422	0.368(0.126–1.078)	0.068	0.100	0.519(0.324–0.831)	6.33 × 10–3	0.786

OR odd ratio, 95%CI confidence interval, P_OR_ P value for the test of association, P_h_ P value for heterogeneity analysis.

**Table 3:** Meta-analysis of the association between *FCGR2B* rs1050501 polymorphism and SLE risk.

Population	N	C vs. T(allele model)			CC vs. CT+TT(recessive model)			CC+CT vs. TT(dominant model)		
		OR(95%CI)	P_OR_	P_h_	OR(95%CI)	P_OR_	P_h_	OR(95%CI)	P_OR_	P_h_
Overall	12	1.236(1.069–1.429)	0.007	0.030	1.754(1.422–2.165)	1.61 × 10^−7^	0.404	1.093(0.952–1.255)	0.205	0.140
Asian	7	1.326(1.095–1.604)	0.006	0.065	1.784(1.408–2.261)	1.67 × 10^−6^	0.630	1.149(0.957–1.380)	0.137	0.121
Caucasian	3	1.087(0.888–1.331)	0.420	0.812	2.055(1.106–3.817)	0.023	0.587	1.019(0.812–1.279)	0.872	0.592
African	1	1.749(1.153–2.655)	0.009	—	2.369(1.198–4.685)	0.013	—	1.777(0.907–3.479)	0.094	—
African-American	1	0.769(0.537–1.099)	0.149	—	0.745(0.353–1.569)	0.438	—	0.733(0.467–1.152)	0.178	—

OR odd ratio, 95%CI confidence interval, P_OR_ P value for the test of association, P_h_ P value for heterogeneity analysis.

**Table 4:** Meta-analysis of the association between FCGR3A rs396991 polymorphism and SLE risk.

Population	N	T vs. G(allele model)			TT vs. TG+GG(recessive model)			TT+TG vs. GG(dominant model)		
		OR(95%CI)	P_OR_	P_h_	OR(95%CI)	P_OR_	P_h_	OR(95%CI)	P_OR_	P_h_
Overall	26	1.17(1.059–1.291)	0.002	0.000	1.263(1.123–1.421)	9.62 × 10^−5^	0.003	1.114(0.933–1.331)	0.232	0.004
Caucasian	9	1.259(1.012–1.566)	0.039	0.000	1.394(1.087–1.789)	9.05 × 10^−3^	0.008	1.187(0.830–1.699)	0.347	0.004
Asian	13	1.152(0.999–1.328)	0.051	0.004	1.211(1.022–1.434)	0.027	0.036	1.164(0.884–1.533)	0.280	0.049
African-American	1	1.022(0.880–1.186)	0.776	—	1.053(0.861–1.287)	0.617	—	0.972(0.712–1.327)	0.858	—
Mixed population	1	1.308(1.029–1.662)	0.028	—	1.585(1.122–2.239)	9.05 × 10^−3^	—	1.172(0.761–1.804)	0.471	—
Non-Caucasian	1	0.903(0.649–1.258)	0.548	—	1.049(0.656–1.677)	0.843	—	0.636(0.336–1.204)	0.164	—
African	1	1.196(0.616–2.324)	0.597	—	1.899(0.638–5.654)	0.249	—	0.750(0.184–3.060)	0.688	—

OR odd ratio, 95%CI confidence interval, P_OR_ P value for the test of association, P_h_ P value for heterogeneity analysis.

**Table 5:** Meta-analysis of the association between FCGR3B copy number polymorphism NA1·NA2 and SLE risk.

Population	N	NA1 vs. NA2(allele model)			NA1·NA1 vs. NA1·NA2+NA2·NA2 (recessive model)			NA1·NA1+NA2·NA2 vs. NA2·NA2 (dominant model)		
		OR(95%CI)	P_OR_	P_h_	OR(95%CI)	P_OR_	P_h_	OR(95%CI)	P_OR_	P_h_
Overall	11	0.851(0.772–0.938)	1.2 × 10^−3^	0.004	0.799(0.685–0.933)	0.005	0.182	0.825(0.702–0.969)	0.019	0.001
Asian	3	0.785(0.697–0.883)	6.07 × 10^−5^	0.040	0.756(0.635–0.898)	0.002	0.116	0.684(0.549–0.853)	7.2 × 10^−4^	0.103
Caucasian	8	1.013(0.851–1.205)	0.888	0.060	1.006(0.709–1.426)	0.974	0.885	1.021(0.806–1.292)	0.866	0.003

OR odd ratio, 95%CI confidence interval, P_OR_ P value for the test of association, P_h_ P value for heterogeneity analysis.

**Table 6:** The allele frequency comparison between the meta-analysis and 1000 Genomes Project.

Polymorphism	Populations	Meta-analysis(alleles frequencies)					
		Cases		Controls		1000 Genomes(Alleles frequencies)	
		A	G	A	G	A	G
SNP rs1801274	Caucasian	0.445	0.555	0.474	0.526	0.500(EUR)	0.5(EUR)
	Asian	0.652	0.348	0.697	0.303	0.722(ASN)	0.278(ASN)
	African	0.568	0.432	0.602	0.398	0.512(AFR)	0.488(AFR)
	African Americans	0.393	0.607	0.529	0.471	0.525(ASW)	0.475(ASW)
	Mixed population	0.479	0.521	0.448	0.552		
	Non-Caucasian	0.493	0.507	0.436	0.564		
	All	0.563	0.437	0.595	0.405	0.57(ALL)	0.43(ALL)
SNP rs1050501		C	T	C	T	C	T
	Asian	0.280	0.720	0.248	0.752	0.255(ASN)	0.745(ASN)
	Caucasian	0.128	0.872	0.107	0.893	0.123(EUR)	0.877(EUR)
	African	0.572	0.428	0.433	0.567	0.248(AFR)	0.752(AFR)
	African-American	0.241	0.759	0.292	0.708	0.213(ASW)	0.787(ASW)
	All	0.249	0.751	0.198	0.802	0.188(ALL)	0.812(ALL)
SNP rs396991		T	G	T	G	T	G
	Caucasian	0.659	0.341	0.629	0.371	0.731(EUR)	0.269(EUR)
	Asian	0.673	0.327	0.657	0.343	0.731(ASN)	0.269(ASN)
	African-American	0.664	0.336	0.659	0.341	0.713(ASW)	0.287(ASW)
	Mixed population	0.622	0.378	0.557	0.443		
	Non-Caucasian	0.600	0.400	0.624	0.376		
	African	0.586	0.414	0.542	0.458	0.785(AFR)	0.215(AFR)
	All	0.663	0.337	0.641	0.359	0.755(ALL)	0.245(ALL)

EUR European ancestry, ASN Asian ancestry, AFR African ancestry, ASW Americans of African Ancestry, ALL All individuals from phase 1 of the 1000 Genomes Project.
