# Supplementary material for: Comprehensive Assessment of the Association between FCGRs polymorphisms and the risk of systemic lupus erythematosus: Evidence from a Meta-Analysis
Source: Sci Rep. 2016 Aug 19;6:31617. doi: 10.1038/srep31617 (PMC4990922; doi:10.1038/srep31617)
Supplement: Supplementary Information [file srep31617-s1.pdf]

Comprehensive Assessment of the Association between *FCGR*s polymorphisms and the risk of systemic lupus erythematosus: Evidence from a Meta-Analysis

Xiao-Wei Zhu[1,2,#], Yong Wang[3,#], Yi-Hua Wei[4,#], Pian-Pian Zhao[1], Xiao-Bo Wang[2], Jing-Jing Rong[1], Wen-Ying Zhong[2], Xing-Wei Zhang[1], Li Wang[1], Hou-Feng Zheng[1,2]

1 Institute of Aging Research, School of Medicine, Hangzhou Normal University, Hangzhou, Zhejiang, China

2 Department of Dermatology, the Affiliated Hospital of Hangzhou Normal University, Hangzhou, Zhejiang, China

3 Department of Cardiovascular Medicine, the Second People's Hospital of Wuhu City, Wuhu, Anhui, China

4 Department of Dermatology, The Affiliated Hospital of Binzhou Medical University, Binzhou, Shandong, China

# Contributed equally to this work.

Corresponding author: Hou-Feng Zheng (hfzheng@hznu.edu.cn) Institute of Aging Research, School of Medicine, Hangzhou Normal University, and The Affiliated Hospital of Hangzhou Normal University, Hangzhou, Zhejiang, China

Supplementary Fig S1a. Forest plot for the meta-analysis of the association between FCGRs polymorphisms and SLE. (FCGR2A rs1801274 and SLE (AA/AG vs GG)

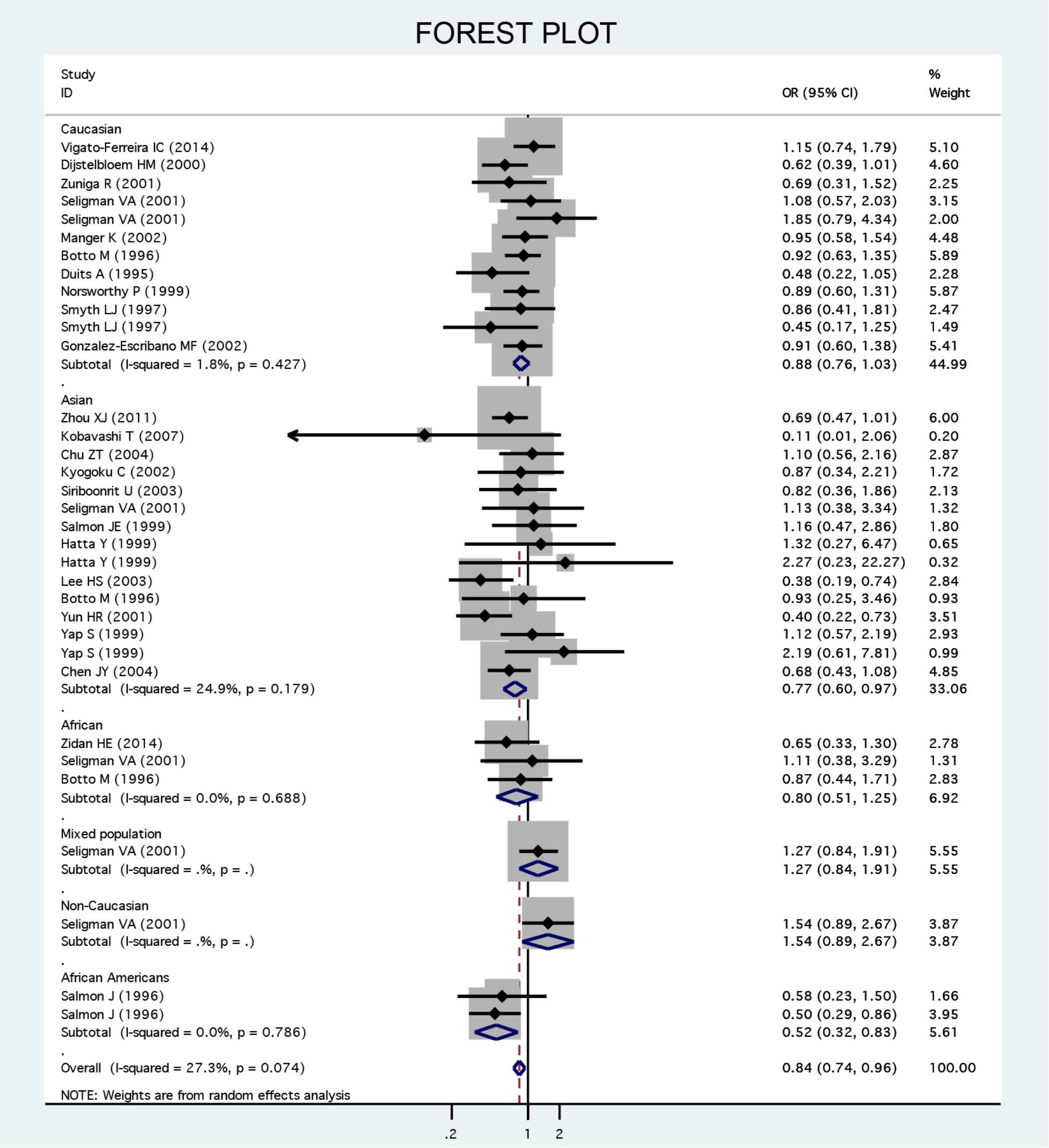

Supplementary Fig S1b. Forest plot for the meta-analysis of the association between FCGRs polymorphisms and SLE. (FCGR2B rs1050501 and SLE (CC/CT vs TT)

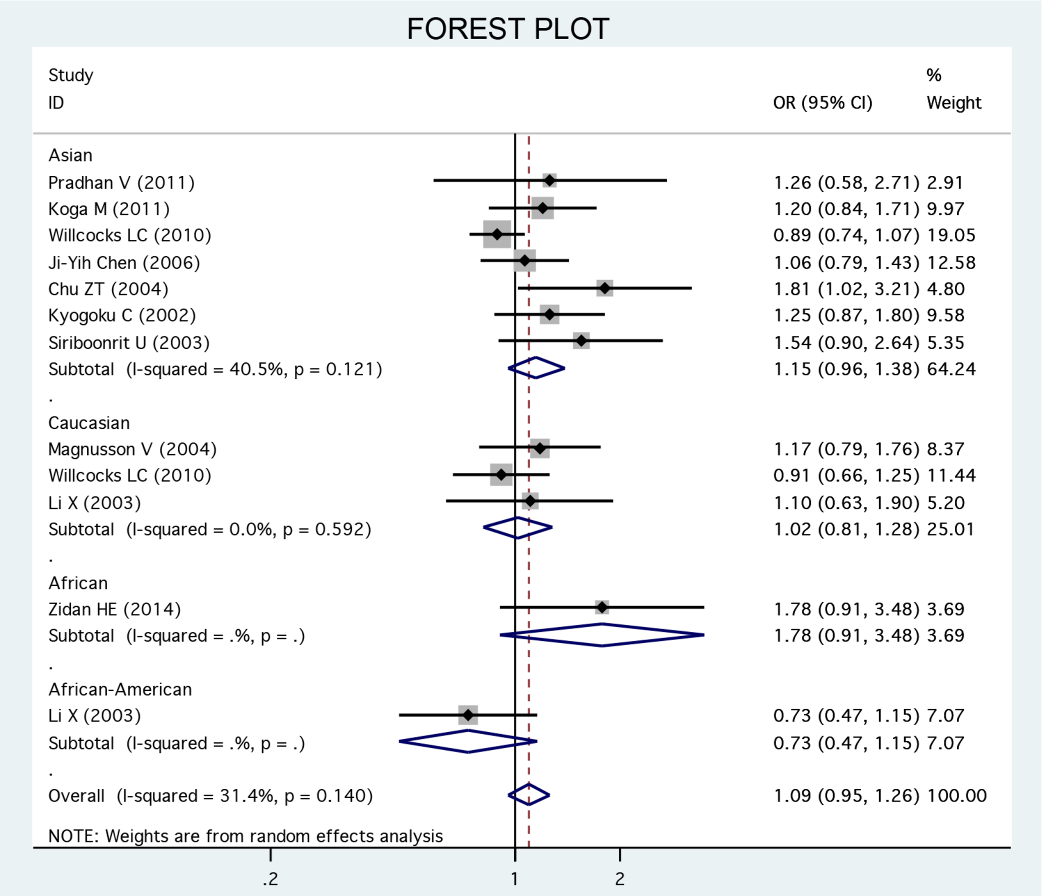

Supplementary Fig S1c. Forest plot for the meta-analysis of the association between FCGRs polymorphisms and SLE. (FCGR3A rs396991 and SLE (TT/TG vs GG)

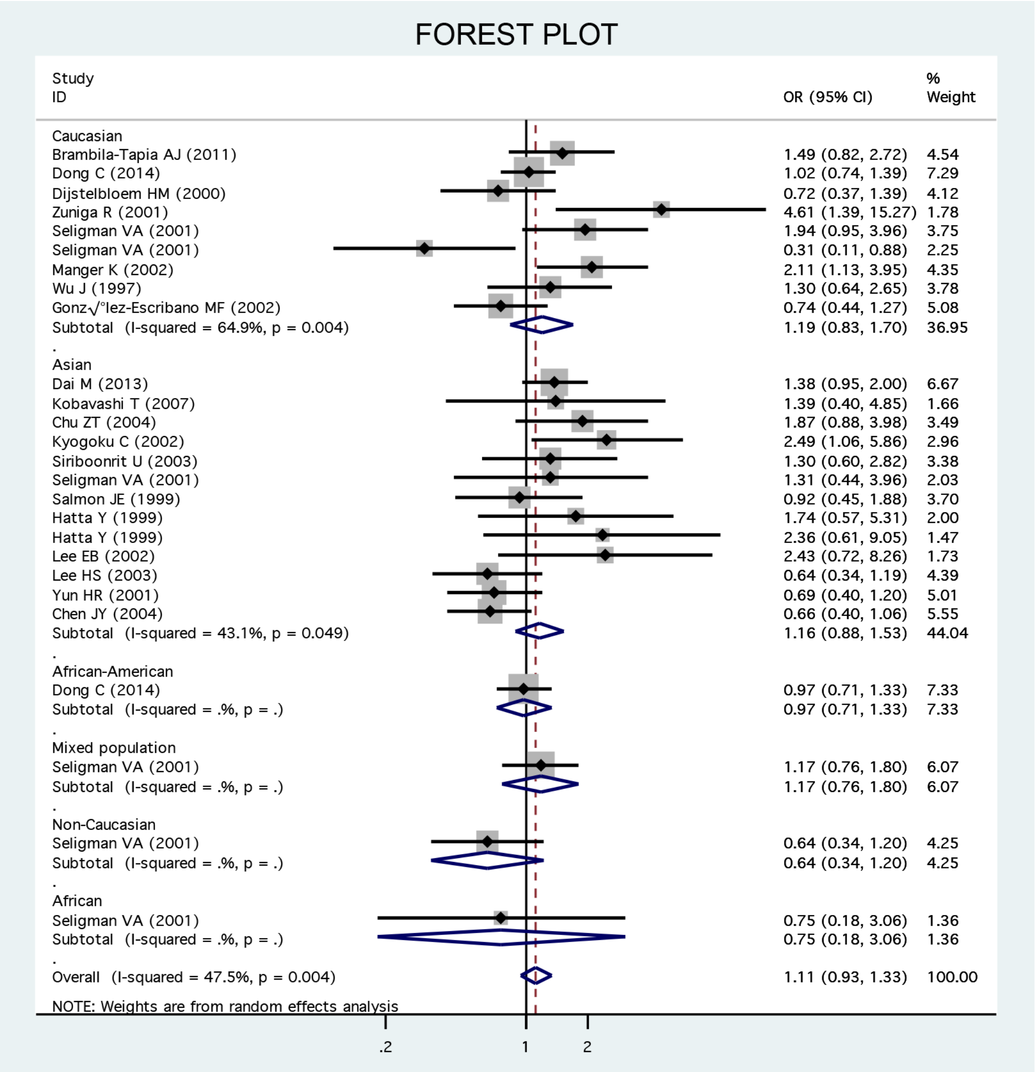

Supplementary Fig S1d. Forest plot for the meta-analysis of the association between FCGRs polymorphisms and SLE. (FCGR3B NA1•NA2 and SLE (NA1 NA1/ NA1 NA2 vs NA2 NA2)

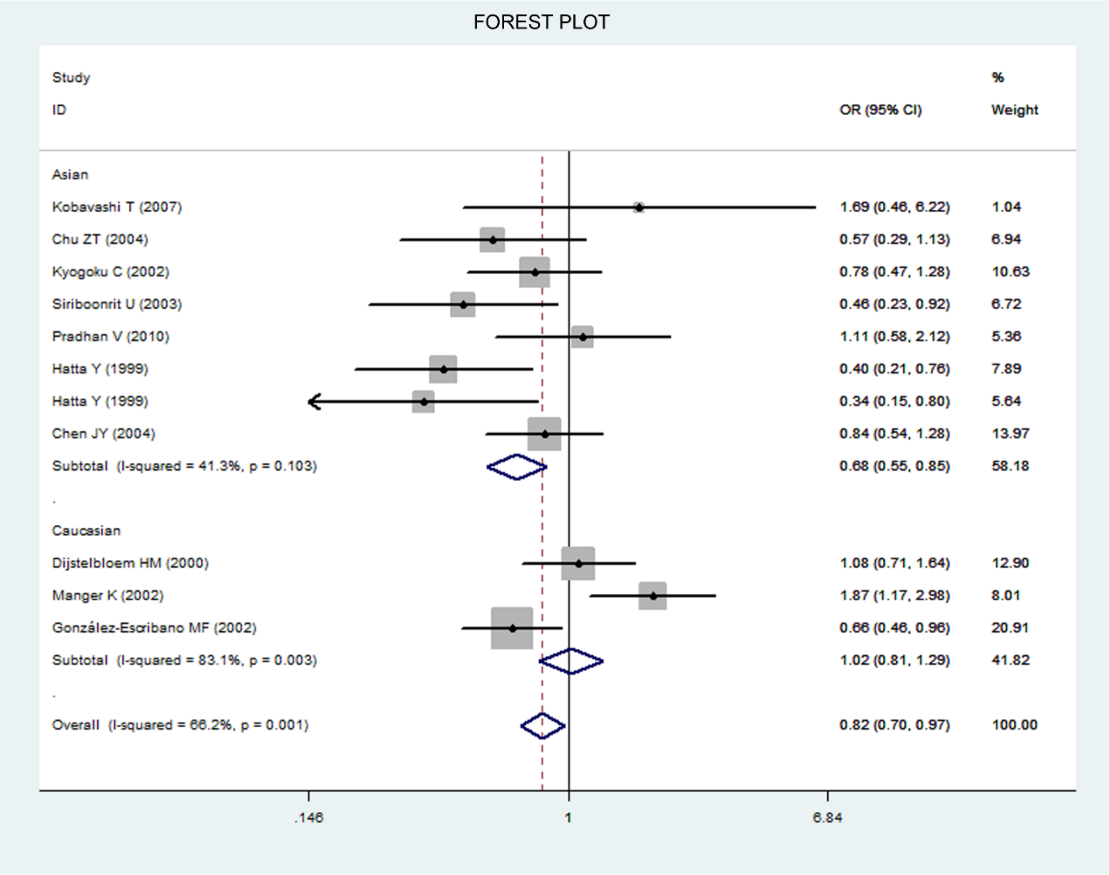

Supplementary Fig S2a. Forest plot for the meta-analysis of the association between FCGRs polymorphisms and SLE. (FCGR2A rs1801274 and SLE (AA vs AG/GG)

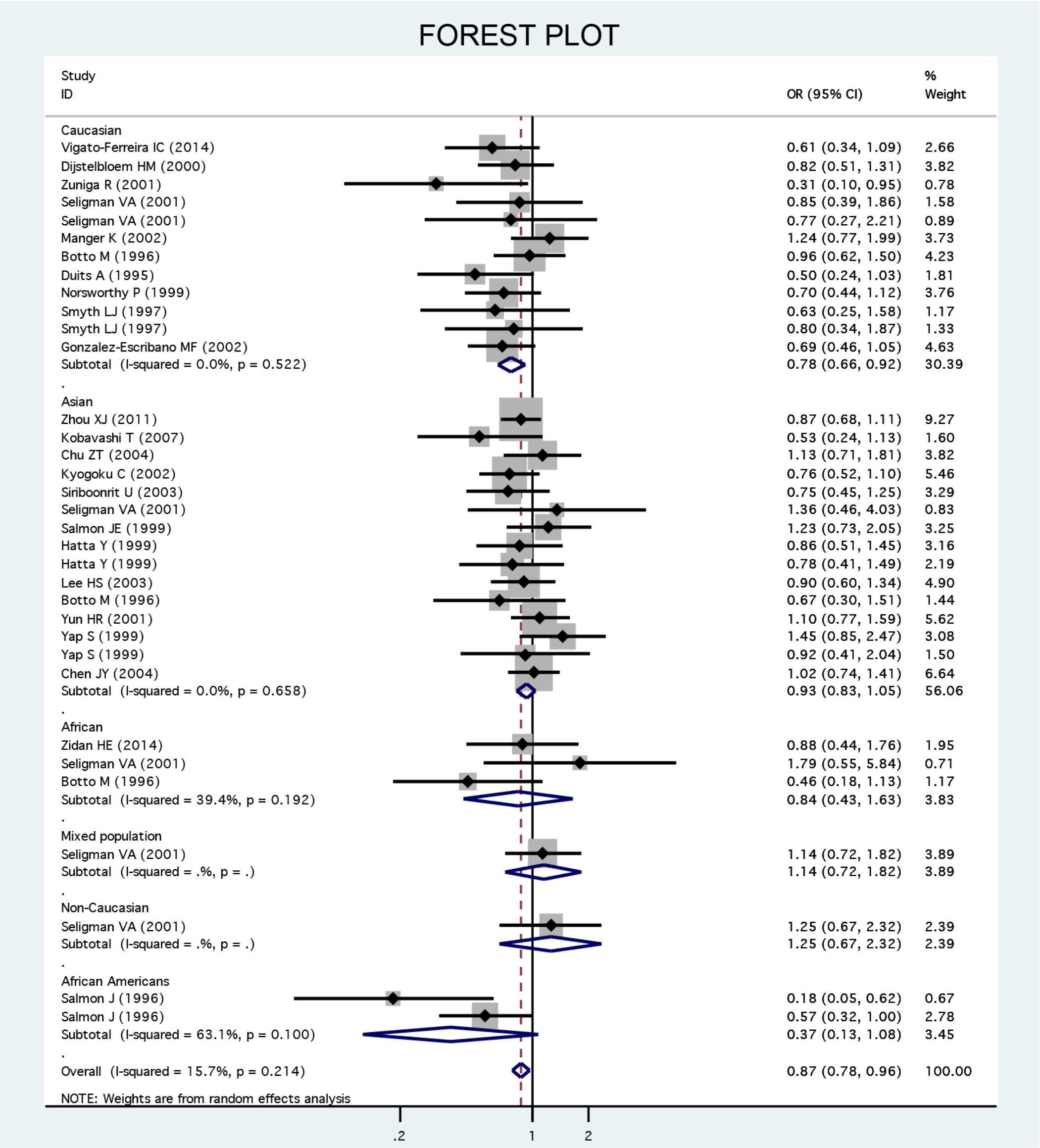

Supplementary Fig S2b. Forest plot for the meta-analysis of the association between FCGRs polymorphisms and SLE. (FCGR2B rs1050501 and SLE (C vs T)

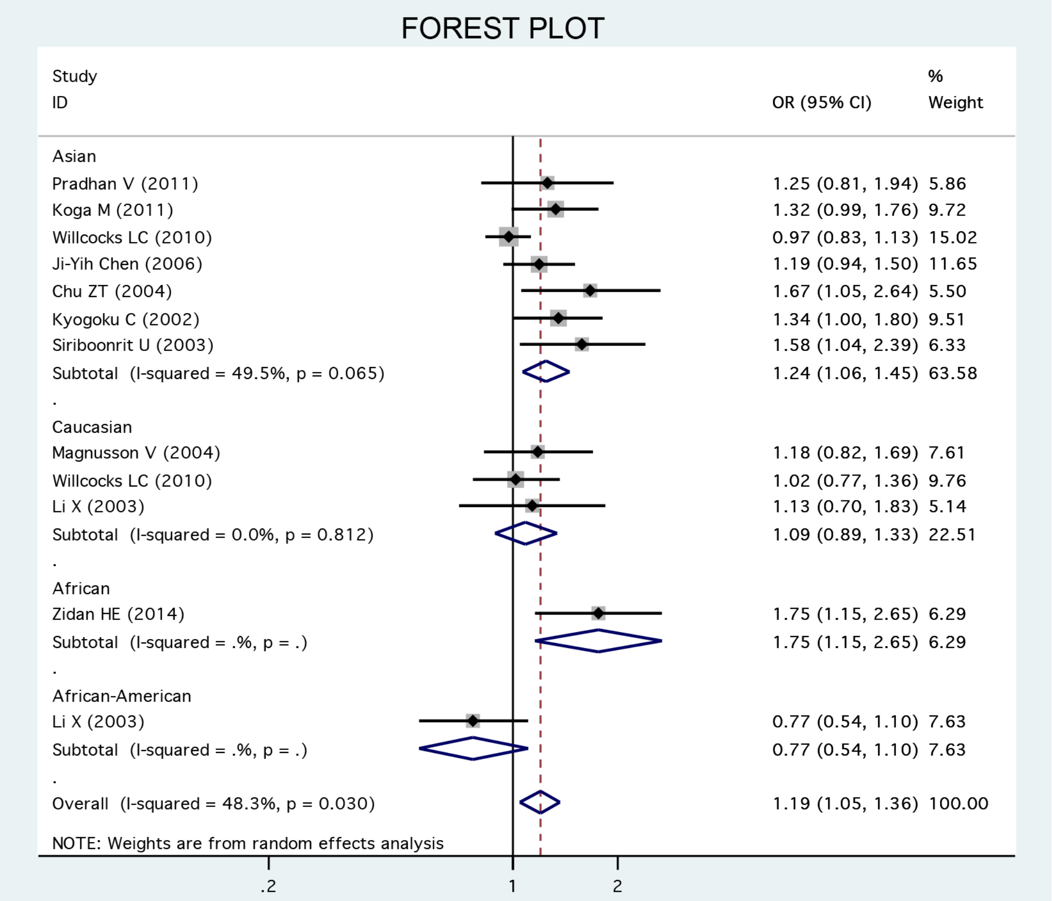

Supplementary Fig S2c. Forest plot for the meta-analysis of the association between FCGRs polymorphisms and SLE. (FCGR3A rs396991 and SLE (T vs G)

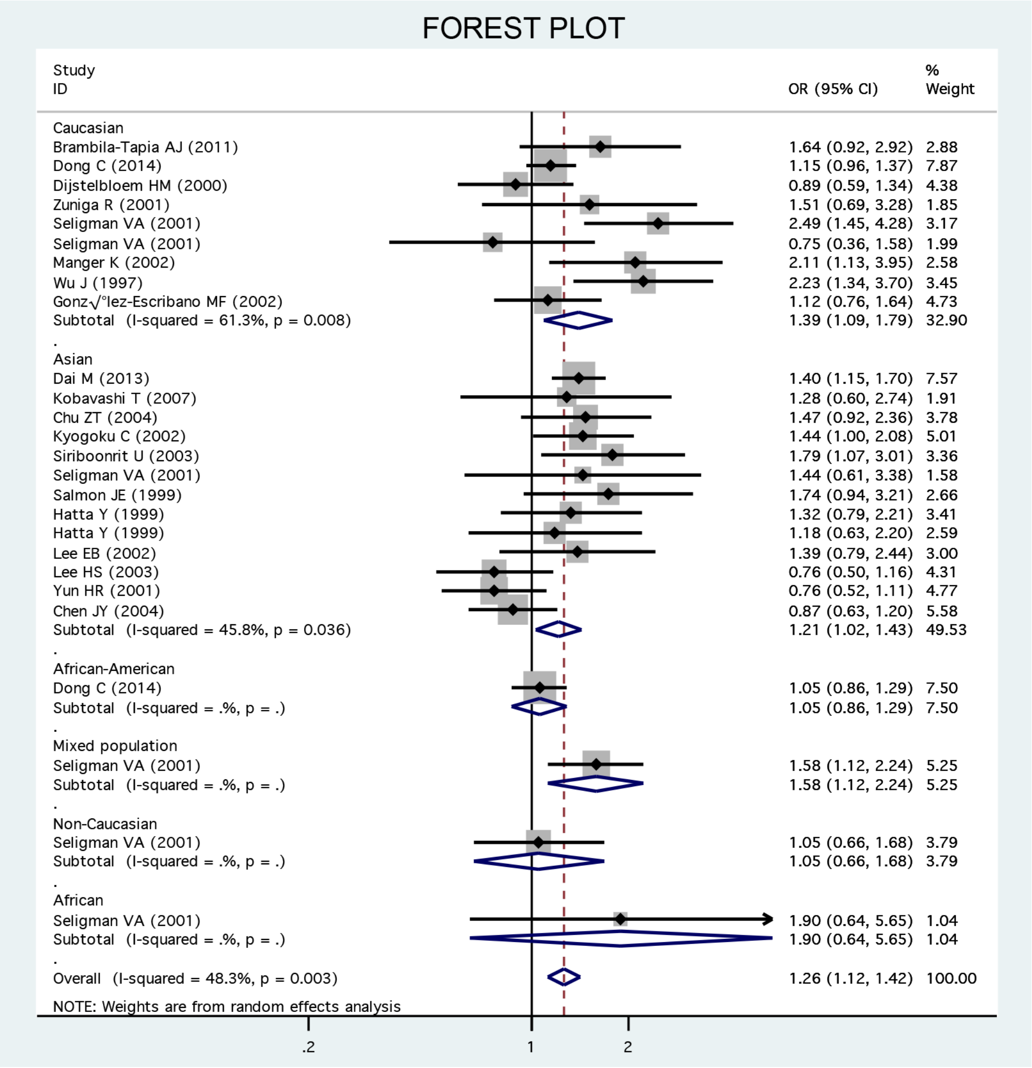

Supplementary Fig S2d. Forest plot for the meta-analysis of the association between FCGRs polymorphisms and SLE. (FCGR3B NA1•NA2 and SLE (NA1NA1 vs NA1NA2/NA2NA2)

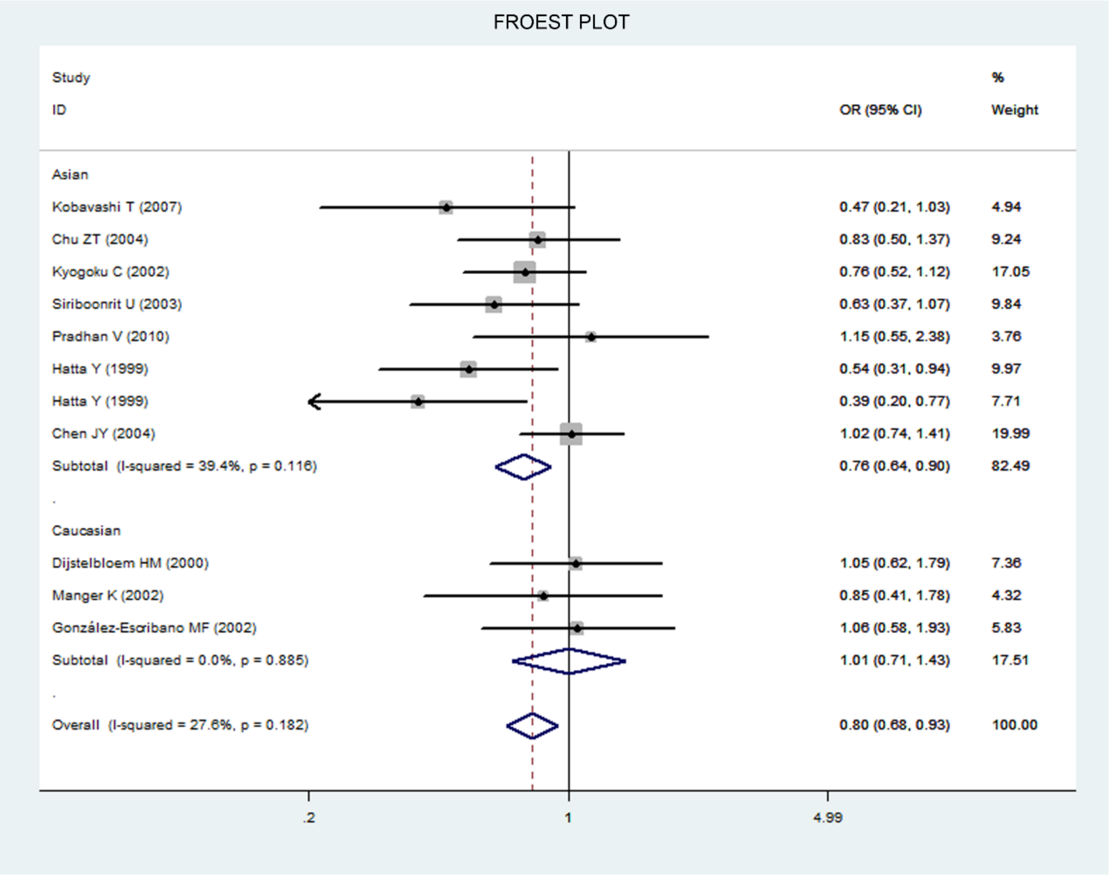

Supplementary Table S1 Begg's Test of publication under three models

	allele model		Recessive model		Dominant model	
	Z	P	Z	P	Z	P
FCGR2A(rs1801274)	0.53	0.594	1.16	0.248	0.77	0.441
FCGR2B(rs1050501)	1.58	0.115	0.21	0.837	1.85	0.064
FCGR3A(rs396991)	1.15	0.252	1.54	0.123	2.07	0.038
FCGR3B(NA1•NA2)	1.25	0.213	0.93	0.350	0.93	0.350
